# Accuracy of biplane x-ray imaging combined with model-based tracking for measuring *in-vivo* patellofemoral joint motion

**DOI:** 10.1186/1749-799X-3-38

**Published:** 2008-09-04

**Authors:** Michael J Bey, Stephanie K Kline, Scott Tashman, Roger Zauel

**Affiliations:** 1 Henry Ford Health Systems, Department of Orthopaedics, Bone and Joint Center; E&R 2015, 2799 W. Grand Blvd, Detroit, MI 48202, USA

## Abstract

**Background:**

Accurately measuring *in-vivo* motion of the knee's patellofemoral (PF) joint is challenging. Conventional measurement techniques have largely been unable to accurately measure three-dimensional, *in-vivo* motion of the patella during dynamic activities. The purpose of this study was to assess the accuracy of a new model-based technique for measuring PF joint motion.

**Methods:**

To assess the accuracy of this technique, we implanted tantalum beads into the femur and patella of three cadaveric knee specimens and then recorded dynamic biplane radiographic images while manually flexing and extending the specimen. The position of the femur and patella were measured from the biplane images using both the model-based tracking system and a validated dynamic radiostereometric analysis (RSA) technique. Model-based tracking was compared to dynamic RSA by computing measures of bias, precision, and overall dynamic accuracy of four clinically-relevant kinematic parameters (patellar shift, flexion, tilt, and rotation).

**Results:**

The model-based tracking technique results were in excellent agreement with the RSA technique. Overall dynamic accuracy indicated errors of less than 0.395 mm for patellar shift, 0.875° for flexion, 0.863° for tilt, and 0.877° for rotation.

**Conclusion:**

This model-based tracking technique is a non-invasive method for accurately measuring dynamic PF joint motion under *in-vivo* conditions. The technique is sufficiently accurate in measuring clinically relevant changes in PF joint motion following conservative or surgical treatment.

## Background

The patellofemoral (PF) joint consists of the distal femur and patella. PF pain syndrome – also known as anterior knee pain or chondromalacia – is very common and is widely believed to be caused by abnormal motion of the patella relative to the femur (often referred to as patellar tracking). Abnormal patellar tracking is thought to alter the mechanical interaction between the patella and femur, and may progress to cartilage degeneration and osteoarthritis.

Accurately measuring *in-vivo* PF joint motion remains a significant challenge. PF joint motion has been measured in cadaver specimens using electromagnetic sensors [1-4], three-dimensional (3D) video analysis of markers [5,6], x-ray stereophotogrammetry [7,8], goniometers [9], and coordinate measuring machines [10]. These studies have provided valuable insight into factors that may influence patellar tracking, but cadaveric experiments are unable to duplicate the *in-vivo* motions, forces, or muscle firing patterns common to live human subjects. *In-vivo* studies of PF joint motion have traditionally relied upon static two-dimensional (2D) radiographs [11-15], 2D video digital fluoroscopy [16], intracortical bone pins [17,18], x-ray photogrammetry [19], electromagnetic sensors [20], static CT [21,22], and static MRI [23-26]. While these studies have also provided helpful information about patellar tracking, static analyses can not quantify PF joint function during dynamic activities, 2D analyses are incapable of capturing the complex 3D relationship of the patella relative to the femur, and bone pins [17,18] limit the number of willing volunteers and make serial studies over time impractical since bone pins can not be reliably reattached in the exact location.

More recently, dynamic MRI-based techniques have grown in popularity as a tool for measuring PF joint motion under *in-vivo* conditions. These techniques – which have been described by various names, including kinematic MRI [27-29], cine phase contrast MRI [30,31], motion-triggered cine MRI [32] or fast phase contrast MRI [33]-acquire a series of MR images as the subject performs a periodic knee motion activity (typically flexion and extension), with each MR image acquired at a unique phase of the knee motion cycle. Thus, multiple motion cycles are required to assemble the MR images necessary to represent a single motion trial. Dynamic MRI techniques that rely upon conventional closed bore scanners are limited by the physical dimensions of the scanner. Specifically, these scanners do not allow for activities that replicate the forces and ranges of motion that produce symptoms for patients with PF pain syndrome. Furthermore, this approach implicitly assumes that there is relatively little variability in knee motion patterns between successive motion cycles.

Additional techniques for assessing *in-vivo* PF joint motion have included dynamic CT imaging [34] and single-plane fluoroscopic imaging combined with shape matching [35]. Dynamic CT imaging has limitations similar to those associated with dynamic MRI. The single-plane fluoroscopic technique is a promising approach that has achieved reasonable levels of theoretical accuracy, but has yet to be validated [35].

To overcome the limitations associated with existing methods for measuring PF joint motion, our laboratory has developed a new model-based tracking technique for measuring *in-vivo* 3D joint motion. The purpose of the study was to assess the accuracy of this model-based tracking technique for *in-vivo* PF joint motion by comparing the model-based technique to an accurate radiostereometric analysis (RSA) technique that measures joint motion by tracking the position of implanted tantalum beads [36].

## Methods

### Overview

We have developed a CT model-based technique for accurately measuring *in-vivo* joint motion from biplane x-ray images. Specific details of this technique, which tracks the position of bones by maximizing the correlation between biplane x-ray images and digitally reconstructed radiographs (DRRs), have been published previously [37]. To validate this new technique, we implanted small beads into the patella and femur of three cadaver knee specimens, recorded biplane radiographic images while manually flexing and extending the leg, measured the position of the patella and femur using model-based tracking, measured the position of the patella and femur with dynamic RSA [36] – our "gold standard" – and then compared the results of the two techniques.

### Specimen preparation

**Figure 1:**
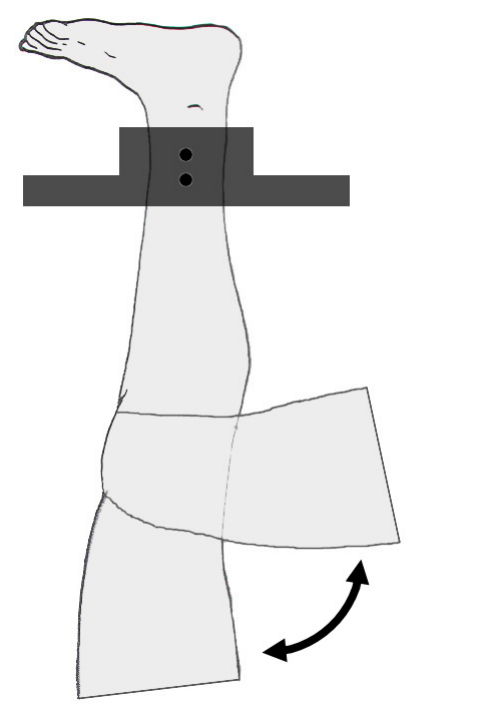
**Experimental testing configuration.** The tibia of each cadaveric leg specimen was rigidly attached to a custom testing fixture, with the leg suspended within the biplane x-ray system in an inverted position. The quadriceps tendon was sutured with nylon cord so that simulated muscle forces could be applied. These manually applied forces flexed the knee from full extension to approximately 80° of flexion at a rate of approximately 60° per second.

Three 1.6 mm diameter tantalum beads were implanted into both the patella and femur of three intact lower limbs from two cadaver specimens (72/male, 89/female). The quadriceps tendon was exposed through a 50 mm skin incision and sutured with nylon cord. The nylon cord was then placed between the skin and quadriceps muscle so that simulated muscle forces could be directed in a physiologic direction parallel to the femur's long axis. The tibia was secured to a custom testing apparatus with the leg inverted, i.e., with the femur hanging passively below the tibia (Figure 1). Although knee flexion is most often accomplished with the tibia rotating relative to a fixed femur, this experimental setup resulted in both the femur and patella moving relative to a fixed tibia and thus resulting in a more challenging assessment of PF joint motion. The specimen was then positioned with the knee centered in a biplane x-ray system [36].

### Testing procedures

Biplane x-ray images were acquired while manually flexing the knee from full extension (i.e., approximately 10° flexion) to approximately 90° of flexion with respect to the femoral and tibial long axes. Knee motion was achieved by manually pulling the nylon cord attached to the quadriceps tendon to cyclically flex and extend the knee. Given that accuracy was assessed by applying both the model-based tracking and dynamic RSA techniques to each trial, it was not necessary to accurately replicate *in-vivo* conditions (i.e., joint motion, muscle forces, or joint contact forces) or insure the repeatability of testing conditions between trials. The biplane x-ray images were acquired at 60 frames per second for 1.5 seconds with the x-ray generators in pulsed mode (70 kV, 320 mA) and video cameras shuttered at 1/500 s to eliminate motion blur. For each specimen, we acquired biplane x-ray images of four flexion-extension trials and two static trials.

Following testing, we obtained axial CT images of each knee using a LightSpeed VCT (GE Medical Systems). The CT data set had 0.625 mm slice spacing and an in-plane resolution of approximately 0.4 mm/pixel. The femur and patella were segmented from surrounding bones and soft tissues (ImageJ 1.32 j, ) and then rescaled with a feature-based interpolation technique that resulted in a 3D bone model with voxel dimensions similar to the biplane x-ray image pixel size.

### Model-based tracking

The 3D positions and orientations of the patella and femur were measured from the biplane x-ray images using a technique referred to as model-based tracking. Briefly, this technique applies a ray-tracing algorithm to project a pair of digitally reconstructed radiographs (DRRs) from the CT-based bone model. The *in-vivo* position and orientation of a bone is estimated by maximizing the correlation between the DRRs and the biplane x-ray images. Using this technique, the 3D position and orientation of the patella and femur were determined independently for all frames of each trial. The final step involved determining the position of the tantalum beads within the CT bone model and then expressing their 3D position relative to a laboratory coordinate system.

### Dynamic RSA

For comparison, the 3D position of each implanted tantalum bead was also determined from the biplane images using a previously validated and well-established dynamic RSA technique [36]. This process determined the 3D location of each implanted tantalum bead relative to the laboratory coordinate system to an accuracy of within ± 0.1 mm. These data enabled a direct comparison with the model-based tracking results.

### Kinematics

PF joint kinematics were determined using transformations between each bone's 3D position and orientation (determined from the model-based tracking and dynamic RSA results) and anatomical axes determined from the CT bone model. Specifically, patellar motion was quantified in terms of shift (i.e., medial-lateral translation relative to the femur), flexion (i.e., rotation about a medial-lateral axis relative to the femur), tilt (i.e., rotation about the patella's long axis), and rotation (i.e., angular position relative to the patella's anterior-posterior axis) [38]. These four parameters are believed to represent the most clinically relevant motion variables. For completeness, anterior-posterior translation and superior-inferior translation of the patella relative to the femur were also measured, even though these two translations are less meaningful from a clinical perspective.

### Comparison of techniques

Accuracy of the model-based tracking technique was quantified in terms of bias and precision [39]. Measurement bias was defined as the average difference between the two techniques. Precision was defined as the standard deviation of the model-based tracking results when applied to only the static trials. Thus, any frame-to-frame variability in measurement error when no motion occurred provided an estimate of the precision of the model-based tracking technique. In addition, to provide a single measurement of accuracy, we assessed the overall dynamic accuracy by calculating the RMS error between the two measurement techniques. These measures of accuracy (i.e., bias, precision, overall dynamic accuracy) were first computed using the 3D position of the implanted tantalum beads as reported by both the model-based and dynamic RSA measurement techniques. This allowed us to assess the amount of error associated with the tracking of each bone. These three measures of accuracy were also calculated for each of the six kinematic measurements (i.e., three translations, three rotations).

## Results

**Figure 2:**
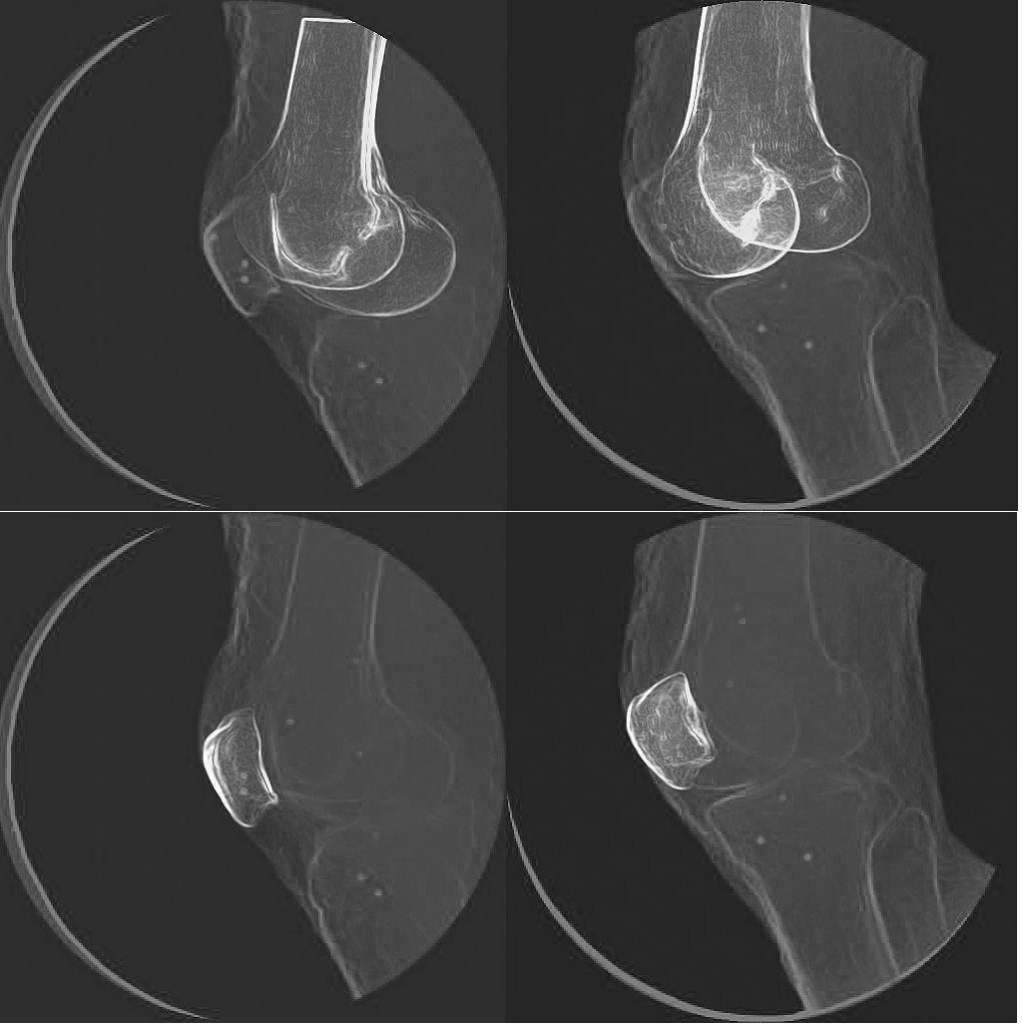
Single-frame model-based tracking solution for the femur (top) and patella (bottom). In each image, the two digitally reconstructed radiographs (DRRs) – i.e., the highlighted bones in each image – are superimposed over the original biplane x-ray images in the position and orientation that maximized the correlation between the DRRs and biplane images.

There was very high agreement between the results from the model-based tracking and RSA techniques (Figure 2). In comparing the position of the implanted tantalum beads, bias ranged from -0.174 to 0.248 mm (depending on coordinate direction), precision ranged from 0.023 to 0.062 mm, and overall dynamic accuracy was better than 0.335 mm (Table 1). When the results were compared using kinematic parameters, bias ranged from -0.293 to 0.320 mm for the three translational parameters (patellar shift, anterior-posterior translation, proximal-distal translation, Table 2) and ranged from -0.090° to 0.475° for the three rotational parameters (flexion, tilt, rotation, Table 2). Precision ranged from 0.042 to 0.114 mm for the three translational parameters and ranged from 0.216° to 0.382° for the three rotational parameters. Overall dynamic accuracy was better than 0.395 mm for the three translational measurements, and better than 0.877° for the rotational measurements (Table 2).

**Table 1:** Accuracy of the model-based technique for tracking the patella and femur was expressed in terms of bias and precision as mean ± standard deviation.

	Bias		Precision		Overall Dynamic Accuracy	
Axis	Patella	Femur	Patella	Femur	Patella	Femur
X	-0.014 ± 0.133	0.207 ± 0.099	0.061 ± 0.027	0.049 ± 0.011	0.220 ± 0.044	0.234 ± 0.064
Y	-0.174 ± 0.114	-0.022 ± 0.125	0.062 ± 0.028	0.038 ± 0.005	0.211 ± 0.035	0.149 ± 0.048
Z	0.248 ± 0.158	0.218 ± 0.099	0.042 ± 0.007	0.023 ± 0.004	0.335 ± 0.127	0.276 ± 0.062

**Table 2:** Accuracy of the model-based technique (RMS errors, mean ± standard deviation) expressed in kinematic parameters that describe motion of the patella relative to the femur.

Kinematic Parameter	Bias	Precision	Overall Dynamic Accuracy
Shift (med/lat translation)	0.320 ± 0.105 mm	0.114 ± 0.039 mm	0.395 ± 0.079 mm
Anterior/posterior translation	-0.293 ± 0.201 mm	0.042 ± 0.011 mm	0.340 ± 0.162 mm
Superior/inferior translation	-0.107 ± 0.312 mm	0.058 ± 0.026 mm	0.315 ± 0.126 mm
Flexion	0.475 ± 0.420°	0.216 ± 0.139°	0.875 ± 0.237°
Tilt	-0.052 ± 0.651°	0.322 ± 0.214°	0.863 ± 0.156°
Rotation	-0.090 ± 0.290°	0.382 ± 0.239°	0.877 ± 0.090°

## Discussion

Accurately measuring PF joint motion is important for understanding, among other things, the effect of conservative and surgical treatment of PF pain syndrome. A previous study that compared patellar tracking patterns between subjects with PF pain and subjects without PF pain reported average differences of approximately 5° in patellar tilt and approximately 4% in patellar offset, i.e., the percentage of the patella lateral to the midline [40]. Assuming an average patellar width of 46 mm [41], this 4% patellar offset corresponds to an estimated difference in patellar translation of approximately 2 mm. Thus, it is reasonable to presume that a system for measuring patellar tracking should be able to detect differences between subject populations in patellar tracking of less than 2 mm and 5°. Using the general rule that a measurement system should ideally have an accuracy that is an order of magnitude better than the smallest change you expect to measure, these data suggest that the patellar tracking technique should have an accuracy of approximately ± 0.2 mm for translations and ± 0.5° for rotations. Although the technique reported here falls short of this ideal accuracy goal, it is still four to five times more accurate than the smallest differences we would hope to detect (i.e., 2 mm of translation and 5° of rotation). From a statistical standpoint, if we assumed that all the variability within a group of subjects was due solely to measurement technique inaccuracy, then the sample size required to detect differences of 2 mm of patellar translation with a measurement system of "ideal" accuracy (i.e., ± 0.2 mm of error) would be 2 subjects (based on a t-test and assuming α = 0.05 and β = 0.2). In contrast, only one additional subject would be required to detect differences of 2 mm with the accuracy of the model-based tracking system reported here (i.e., ± 0.395 mm). However, since previously reported data indicates that inter-subject variability in measured knee kinematics is approximately 10 to 30 times greater than the inaccuracies associated with the measurement system reported here [40], the authors are comfortable that the technique reported here is still within an acceptable accuracy range for detecting clinically significant differences in PF joint motion.

Although a number of techniques for measuring *in-vivo* PF joint motion have been previously reported, the accuracy of these techniques is reported far less frequently. For example, Rebmann and Sheehan compared three cine phase contrast MR imaging protocols for measuring *in-vivo* knee kinematics in terms of precision and subject inter-exam variability, but did not report any explicit measures of accuracy [33]. Similarly, Powers and colleagues have published extensively on PF joint motion and have presented measures of repeatability [28,42], but the authors are not aware of any report that explicitly describes the 3D accuracy of their MRI-based measurement technique. Although these measures of repeatability provide some insight into the suitability of a measurement technique – especially in contrast to studies that fail to report any measures of accuracy or reliability [32,34,43] – it is important to remember that repeatability should not be confused with accuracy. Systematic errors can cause poor data accuracy, but would not necessarily affect repeatability.

In contrast, several authors have carefully determined the accuracy of their techniques for measuring *in-vivo* PF joint motion. For example, Sheehan and colleagues used a gear-driven phantom object to assess the 3D accuracy of cine phase contrast MRI for measuring joint motion [30,31]. These data indicated average absolute tracking errors of less than ± 0.7 mm for in-plane motions, and slightly higher (up to 1.8 mm) of error for out-of-plane motions. Fregly and colleagues provided a rigorous theoretical accuracy assessment model-based tracking technique applied to single-plane fluoroscopic images [35]. The authors reported good measures of accuracy (e.g., bias less than 0.75 mm and 0.4°, though precision as high as ± 4 mm and ± 1.8°) with their flat-shading technique. However, these values are from a theoretical study where all other sources of error were eliminated and it is not yet known if this level of accuracy can be achieved under experimental conditions.

**Figure 3:**
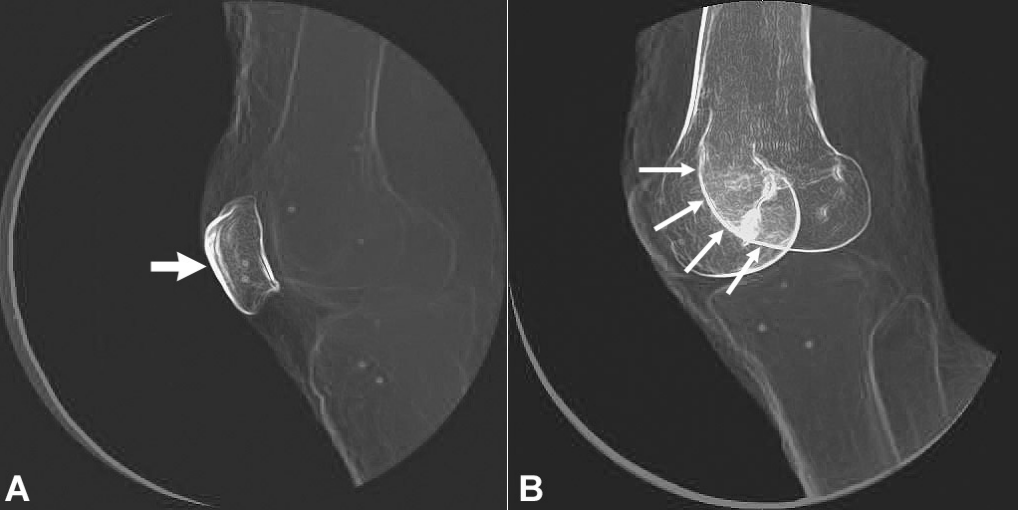
**The model-based tracking technique relies upon:** A) internal information such as subtle differences in bone density and/or B) the presence of bone edges in an x-ray image that do not necessarily contribute to the outline of a particular bone in all joint positions.

We believe that it is necessary to conduct a validation study for each anatomical joint to which we intend to apply the model-based tracking technique. Stated another way, we believe that it would be highly inappropriate to validate this technique for, say, the glenohumeral joint and then assume that the accuracy levels obtained in that particular validation study could be assumed to be the same for every other anatomical joint. This belief is based on the fact that the factors influencing the accuracy of the model-based technique are not the same for all anatomical joints, and that the conditions for conducting validation studies should as much as possible resemble actual *in-vivo* testing conditions. The specific factors influencing the accuracy of this technique include the 3D shape of a particular bone, the amount of "internal" bone information (i.e., variability in bone density and/or the presence of bone edges that appear in an x-ray image but do not necessarily contribute to the outline of a particular bone in all joint positions, Figure 3), the presence of surrounding soft tissues, overlap from surrounding bones, the magnitude of joint motion, and the velocity of joint motion. Although we have not yet assessed the relative influence of each of these factors to model-based tracking accuracy, this list of factors comes from first-hand experience with the technique.

The advantages of this technique of combining model-based tracking with biplane x-ray imaging is that it provides accurate, 3D, non-invasive measures of PF joint motion during functional activities that are known to produce symptoms for patients diagnosed with PF pain syndrome (e.g., normal gait, stair climbing/descending). There are two primary disadvantages to this technique. The first is the that the amount of x-ray exposure associated with the CT scan and biplane x-ray imaging limits the number of trials that can be performed. However, all testing procedures have been approved by both the Institutional Review Board and the Radiation Safety Committee at Henry Ford Hospital. The second disadvantage is that the field of view is limited to the biplane x-ray system's 3D imaging volume, i.e., the region defined by the intersecting x-ray beams. Although this limitation prevents us from collecting biplane x-ray images during an entire gait cycle, we still can collect information for the vast majority of the stance phase when the muscle forces, joint forces, and pain are the highest. Another limitation of this study is that accuracy of this measurement technique was not explicitly assessed at knee flexion angles greater than approximately 90°.

In summary, this model-based tracking approach is a non-invasive technique for accurately measuring *in-vivo* PF joint motion during dynamic activities. The results indicate that model-based tracking can measure *in-vivo* motion of the patella to within 0.455 mm and 0.987°. The technique achieves a level of accuracy that is necessary and sufficient for addressing clinically relevant questions regarding PF joint function. Future research will use this technique to analyze the effects of conservative and surgical treatment of PF pain syndrome.

## Competing interests

The authors declare that they have no competing interests.

## Authors' contributions

MJB designed this study, participated in the data collection and analysis, and drafted the manuscript. SKK participated in the data collection and analysis. ST participated in study design and data analysis. RZ developed the data analysis software. All authors read and approved the final manuscript.

## References

[B1] Hsu HC, Luo ZP, Rand JA, An KN (1997). Influence of lateral release on patellar tracking and patellofemoral contact characteristics after total knee arthroplasty. J Arthroplasty.

[B2] Hefzy MS, Jackson WT, Saddemi SR, Hsieh YF (1992). Effects of tibial rotations on patellar tracking and patello-femoral contact areas. J Biomed Eng.

[B3] Chew JT, Stewart NJ, Hanssen AD, Luo ZP, Rand JA, An KN (1997). Differences in patellar tracking and knee kinematics among three different total knee designs. Clin Orthop Relat Res.

[B4] Sakai N, Luo ZP, Rand JA, An KN (1996). *In vitro* study of patellar position during sitting, standing from squatting, and the stance phase of walking. Am J Knee Surg.

[B5] Goh JC, Lee PY, Bose K (1995). A cadaver study of the function of the oblique part of vastus medialis. J Bone Joint Surg Br.

[B6] Sandmeier RH, Burks RT, Bachus KN, Billings A (2000). The effect of reconstruction of the medial patellofemoral ligament on patellar tracking. Am J Sports Med.

[B7] Heegaard J, Leyvraz PF, Van Kampen A, Rakotomanana L, Rubin PJ, Blankevoort L (1994). Influence of soft structures on patellar three-dimensional tracking. Clin Orthop Relat Res.

[B8] van Kampen A, Huiskes R (1990). The three-dimensional tracking pattern of the human patella. J Orthop Res.

[B9] Ahmed AM, Duncan NA, Tanzer M (1999). *In vitro* measurement of the tracking pattern of the human patella. J Biomech Eng.

[B10] Kwak SD, Ahmad CS, Gardner TR, Grelsamer RP, Henry JH, Blankevoort L, Ateshian GA, Mow VC (2000). Hamstrings and iliotibial band forces affect knee kinematics and contact pattern. J Orthop Res.

[B11] Bockrath K, Wooden C, Worrell T, Ingersoll CD, Farr J (1993). Effects of patella taping on patella position and perceived pain. Med Sci Sports Exerc.

[B12] Larsen B, Andreasen E, Urfer A, Mickelson MR, Newhouse KE (1995). Patellar taping: a radiographic examination of the medial glide technique. Am J Sports Med.

[B13] Merchant AC, Mercer RL, Jacobsen RH, Cool CR (1974). Roentgenographic analysis of patellofemoral congruence. J Bone Joint Surg Am.

[B14] Sikorski JM, Peters J, Watt I (1979). The importance of femoral rotation in chondromalacia patellae as shown by serial radiography. J Bone Joint Surg Br.

[B15] Tyler TF, Hershman EB, Nicholas SJ, Berg JH, McHugh MP (2002). Evidence of abnormal anteroposterior patellar tilt in patients with patellar tendinitis with use of a new radiographic measurement. Am J Sports Med.

[B16] Stein LA, Endicott AN, Sampalis JS, Kaplow MA, Patel MD, Mitchell NS (1993). Motion of the patella during walking: a video digital-fluoroscopic study in healthy volunteers. AJR Am J Roentgenol.

[B17] Lafortune MA, Cavanagh PR, Sommer HJ, Kalenak A (1992). Three-dimensional kinematics of the human knee during walking. J Biomech.

[B18] Koh TJ, Grabiner MD, De Swart RJ (1992). *In vivo* tracking of the human patella. J Biomech.

[B19] Veress SA, Lippert FG, Hou MC, Takamoto T (1979). Patellar tracking patterns measurement by analytical x-ray photogrammetry. J Biomech.

[B20] Laprade J, Lee R (2005). Real-time measurement of patellofemoral kinematics in asymptomatic subjects. Knee.

[B21] Schutzer SF, Ramsby GR, Fulkerson JP (1986). The evaluation of patellofemoral pain using computerized tomography. A preliminary study. Clin Orthop Relat Res.

[B22] Pinar H, Akseki D, Genc I, Karaoglan O (1994). Kinematic and dynamic axial computerized tomography of the normal patellofemoral joint. Knee Surg Sports Traumatol Arthrosc.

[B23] Fellows RA, Hill NA, Gill HS, MacIntyre NJ, Harrison MM, Ellis RE, Wilson DR (2005). Magnetic resonance imaging for in vivo assessment of three-dimensional patellar tracking. J Biomech.

[B24] Hinterwimmer S, von Eisenhart-Rothe R, Siebert M, Welsch F, Vogl T, Graichen H (2004). Patella kinematics and patello-femoral contact areas in patients with genu varum and mild osteoarthritis. Clin Biomech (Bristol, Avon).

[B25] Kujala UM, Osterman K, Kormano M, Komu M, Schlenzka D (1989). Patellar motion analyzed by magnetic resonance imaging. Acta Orthop Scand.

[B26] von Eisenhart-Rothe R, Siebert M, Bringmann C, Vogl T, Englmeier KH, Graichen H (2004). A new *in vivo* technique for determination of 3D kinematics and contact areas of the patello-femoral and tibio-femoral joint. J Biomech.

[B27] Powers CM, Ward SR, Fredericson M, Guillet M, Shellock FG (2003). Patellofemoral kinematics during weight-bearing and non-weight-bearing knee extension in persons with lateral subluxation of the patella: a preliminary study. J Orthop Sports Phys Ther.

[B28] Powers CM, Shellock FG, Pfaff M (1998). Quantification of patellar tracking using kinematic MRI. J Magn Reson Imaging.

[B29] Shellock FG, Mink JH, Deutsch AL, Foo TK, Sullenberger P (1993). Patellofemoral joint: identification of abnormalities with active-movement, "unloaded" versus "loaded" kinematic MR imaging techniques. Radiology.

[B30] Sheehan FT, Zajac FE, Drace JE (1998). Using cine phase contrast magnetic resonance imaging to non-invasively study in vivo knee dynamics. J Biomech.

[B31] Sheehan FT, Zajac FE, Drace JE (1999). *In vivo* tracking of the human patella using cine phase contrast magnetic resonance imaging. J Biomech Eng.

[B32] Brossmann J, Muhle C, Schroder C, Melchert UH, Bull CC, Spielmann RP, Heller M (1993). Patellar tracking patterns during active and passive knee extension: evaluation with motion-triggered cine MR imaging. Radiology.

[B33] Rebmann AJ, Sheehan FT (2003). Precise 3D skeletal kinematics using fast phase contrast magnetic resonance imaging. J Magn Reson Imaging.

[B34] Dupuy DE, Hangen DH, Zachazewski JE, Boland AL, Palmer W (1997). Kinematic CT of the patellofemoral joint. AJR Am J Roentgenol.

[B35] Fregly BJ, Rahman HA, Banks SA (2005). Theoretical accuracy of model-based shape matching for measuring natural knee kinematics with single-plane fluoroscopy. J Biomech Eng.

[B36] Tashman S, Anderst W (2003). *In-vivo* measurement of dynamic joint motion using high speed biplane radiography and CT: application to canine ACL deficiency. J Biomech Eng.

[B37] Bey MJ, Zauel R, Brock SK, Tashman S (2006). Validation of a new model-based tracking technique for measuring three-dimensional, *in vivo* glenohumeral joint kinematics. J Biomech Eng.

[B38] Bull AM, Katchburian MV, Shih YF, Amis AA (2002). Standardisation of the description of patellofemoral motion and comparison between different techniques. Knee Surg Sports Traumatol Arthrosc.

[B39] ASTM (1996). Standard Practice for Use of the Terms Precision and Bias in ASTM Test Methods.

[B40] Powers CM (2000). Patellar kinematics, part II: the influence of the depth of the trochlear groove in subjects with and without patellofemoral pain. Phys Ther.

[B41] Baldwin JL, House CK (2005). Anatomic dimensions of the patella measured during total knee arthroplasty. J Arthroplasty.

[B42] Ward SR, Shellock FG, Terk MR, Salsich GB, Powers CM (2002). Assessment of patellofemoral relationships using kinematic MRI: comparison between qualitative and quantitative methods. J Magn Reson Imaging.

[B43] Muhle C, Brossmann J, M. H (1996). Kinematic MRI of the knee using a specially designed positioning device. J Comput Assist Tomogr.

